# Deep Vein Thrombosis and the Neurosurgical Patient

**DOI:** 10.21315/mjms2019.26.5.13

**Published:** 2019-11-04

**Authors:** Rakesh Rethinasamy, Azmi Alias, Regunath Kandasamy, Azman Raffiq, Mun Choon Looi, Tassha Hillda

**Affiliations:** 1 Hospital Kuala Lumpur, Kuala Lumpur, Malaysia; 2 School of Medical Sciences, Hospital Universiti Sains Malaysia, Kubang Kerian, Kelantan, Malaysia

**Keywords:** deep vein thrombosis, trauma, brain tumour, Well’s score, D-dimer

## Abstract

**Background:**

Neurosurgical patients are varied, encompassing cranial and spinal diseases and trauma, and are admitted under both elective and emergency settings. In all settings, neurosurgery patients are at risk of deep vein thrombosis. D-dimer and ultrasound Doppler have long been good screening and confirmatory tools for the diagnosis of deep vein thrombosis (DVT). We conducted a study to identify the factors associated with DVT among neurosurgical patients, and the overall rate of occurrence at our centre. We aimed to also compare our results to the incidence in similar studies elsewhere in which more judicious use of pharmacological prophylaxis was undertaken. We also included the Well’s score to validate its usefulness in screening for DVT in our local setting.

**Methods:**

All patients admitted into our centre were screened for eligibility and those who underwent surgery from September 2016 to September 2017 had a D-dimer screening after surgery, followed by an ultrasound Doppler if the former was positive. The choice of anticoagulant therapy was not influenced by this study, and observation of the use was in keeping with usual practices in our centre was done.

**Results:**

A total number of 331 patients were recruited in this study, however, after the inclusion and exclusion criteria had been met, 320 patients remained eligible, i.e. suitable for analysis. The mean age of our patients was 46 years, with 66% being male patients. A majority of the cases in this study were cranial related, with only 5% being spine surgeries. On the multivariate analysis, the Well’s score and the number of days in bed remained statistically significant, after adjusting for age group, gender, ethnicity, type of central venous access and type of DVT prophylaxis with an adjusted odd’s ratio, and a confidence interval of 95%, and *P* < 0.05 for each.

**Conclusion:**

Well’s scoring and number of days in bed were independent factors affecting the rate of DVT in patients undergoing neurosurgical procedures in our centre.

## Introduction

Venous thromboembolism is a significant health issue on a global scale, affecting a wide variety of people, for an equally wide variety of reasons. Deep vein thrombosis (DVT) is the formation of clots in the deep venous system of the body and primarily affects the large veins in the lower leg, thighs, and can also occur in deep veins of the arms and pelvis.

The development of DVT is a constant risk in any hospital setting. Although most DVTs are occult and can resolve spontaneously, complications from it arises when the clot dislodges and blocks narrower veins of major organs such as the heart, lung and brain, which can result in catastrophic outcome. Prophylaxis of DVT is therefore an integral as part of any patient care.

The pathophysiology of DVT is described in detail elsewhere, with Virhow’s triad-hypercoagulability, stasis and endothelial damage, forming the basis of development of the disease process. A multitude of risk factors such as oral contraceptive pills, obesity, prolonged bed rest and immobility contribute to the added chances of developing DVT. Certain medical conditions such as malignancies and Factor V Leiden have also been shown to increase the risk of developing DVT (1, 2). DVT can spontaneously resolve, however, where there is no resolution, the clot itself can dislodge and cause life threatening complications such as pulmonary embolism and cranial infarct.

The reported incidences of DVT in neurosurgical patients are varied and range from 12.02% to 26.1% (3, 4). The risk of DVT development in a hospital setting can be reduced with the administration of anticoagulant prophylaxis. Given however, for many of the neurosurgical patients, the potential complication of developing a new bleed or worsening of an existing bleed, with catastrophic consequences, often discourages the neurosurgeon from regular use of anticoagulants. One study involving trauma patients showed a 13-fold increase risk of enlarging clot size (5). As such, there are no clear guidelines for the use of anticoagulants in neurosurgical patients, with practices varying from centre to centre. The usual practice in our centre for the prophylaxis of DVT remains mostly mechanical, either in the form of pneumatic cuffs, or TED stockings. Patients were encouraged to ambulate at the earliest possible time as part following surgery. One further common challenge was in defining a recommended D-dimer value for the screening of DVT using D-dimer, as the proposed upper limits were not consistent in the settings of the normal population versus differing inpatients.

In this study, we aimed to identify the risk factors associated with the development of DVT in neurosurgical patients, with the objective of being able to identify types of patients with the highest risks in our given cohort and therefore, to assist the neurosurgeon in deciding on patients who would benefit from more aggressive coverage with anticoagulants. We had also included Well’s scoring to determine its validity in an inpatient neurosurgical setting as it was not a routine tool used for our patient assessment. We further looked at D-dimer values to compare with the limits that had been suggested in other studies.

## Methods

### Research Design

This is a prospective cohort study using non-probability sampling to determine the factors that are associated with, and the overall rate of, DVT among neurosurgery patients who have been admitted and subjected to surgery in Kuala Lumpur Hospital, between September 2016 to September 2017. The patient population, including those admitted under both elective and emergency settings, had undergone at least one neurosurgical procedure during that admission. No additional deep vein thrombosis prophylaxis was given for patients for the purpose of this study.

### Inclusion and Exclusion Criteria

#### Inclusion

- Patients aged between 18–65 years
- At least one neurosurgical procedure done during admission

#### Exclusion

- Patients on anticoagulants, or a past history of venous thrombosis
- Bed or wheelchair bound for more than 3 days prior to admission
- Patients who were unable to give consent themselves, or via the next of kin

All patients who were selected after applying the inclusion and exclusion criteria underwent a D-dimer (Hemosil D-dimer IL ACL7000) screening three days after surgery. Using a screening value suggested in a study by Prell et al., (6) a cut of value of 2 mg/L was used. Patients who had values exceeding 2 mg/L were then subjected to an ultrasound Doppler of both the lower limbs to rule out deep vein thrombosis. All ultrasonography was performed by or under supervision of a qualified radiologist. If DVT was diagnosed, medical treatment was commenced and the patients were followed up until the resolution of DVT. For patients who did not have an elevated D-dimer value, they were excluded from undergoing ultrasound Doppler unless clinical suspicion arose during the admission period. All patients were also clinically assessed for calf tenderness and swelling throughout their hospital stay. Detailed data collection documenting demographics, physical examination, Well’s Scoring, diagnosis and surgical procedures were recorded.

### Data Collection and Analysis

The data we collected was keyed into the computer software Statistical Package for Social Sciences (SPSS) for Mac version 24.0. The data generally contained demographic information, DVT risk factors, surgical details, D-dimer as well as ultrasound Doppler findings. Demographic information was expressed in a table form as mean and standard deviation for numerical variables and number and percentage for categorical variable. The predictors of DVT were analysed with univariate logistic regression and multiple logistic regression to report on crude and adjusted respectively.

## Results

A total number of 331 patients were recruited in this study, however, after the inclusion and exclusion criteria were met, 320 patients remained eligible i.e. suitable for analysis. The patients’ demographics are depicted in Table 1. Mean age (SD) was 46 years, with the youngest being 18, and eldest being 64. A majority of our patients were from the Malay ethnicity (*n* = 204), followed by Chinese (*n* = 82), Indians (*n* = 25) and lastly others (*n* = 9). This reflects a gross general population mix for the region. There were nearly twice as many males (*n* = 212) in our study compared to females (*N* = 108). The majority of cases were cranial (*n* = 304), the rest being spine surgeries (*n* = 16).

Most of the cases also came under emergency settings (*n* = 242). We further divided patients according to the Well’s Score, into low risk (*n* = 280) if the score was less than 3 and high risk (*n* = 40) if the score was 3 or more. The choice of DVT prophylaxis among our subjects were compression stockings (*n* = 125), pneumatic cuff (*n* = 166), low molecular weight heparin (*n* = 23), while the remainder received none (*n* = 6). Most of the central venous access obtained in our patients were femoral (*n* = 233), followed by subclavian (*n* = 38), and jugular (*n* = 1) with the remainder not having any at all (*n* = 48).

Our study subjected all patients to a D-dimer screening, of which over one third (*n* = 133) had results of more than 2 mg/L, with the remainder (*n* = 187) having results less than the chosen cut off value. From those who had been subjected to ultrasound Doppler, a total of 33 were confirmed to have DVT. This gave us an overall DVT rate of 10.3%, with a 95% confidence interval, 7%–13.6%.

Univariate logistic regression (with 1 to 1 association) was done for the characteristics of patients as a predictive indicator for development of DVT as shown in Table 2. Type of central venous access (*P* < 0.001), type of DVT prophylaxis (*P* < 0.014), increased number of days in bed (*P* < 0.029) and Well’s score (*P* < 0.006) had significantly higher odds of developing DVT.

On the multivariate analysis, as depicted in Table 3, the Well’s Score and the number of days in bed remained statistically significant after adjusting for age group, gender, ethnicity, type of central venous access and type of DVT prophylaxis. The results showed that those within the high risks group of the Well’s scoring had an increased risk of developing DVT by 7.2 times. With regards to the number of days in bed, a patient with an increase of 1 day in bed has 1.02 times the odds of developing DVT (95% CI, 1.002–1.05, *P* = 0.029). Type of tumour was removed from the regression since there was only 1 observation of spinal tumour in positive diagnosis, making a comparison of rate between cranial and spinal neurosurgical tumour cases non-feasible.

## Discussion

DVT remains a common and potentially life-threatening complication in any inpatient setting. The incidence of DVT in neurosurgical centres can vary significantly from 7.9% to 29% (7, 8). In many neurosurgical centres, there remains some ambiguity in the guidelines and practice for the use of pharmacological prophylaxis of DVT. In our centre, prophylaxis for DVT is mostly mechanical, either in the form of intermittent pneumatic cuffs or compression stockings. The diagnosis of DVT is confirmed with an ultrasound Doppler after clinical suspicion warrants a D-dimer screening. Patients are screened when they develop calf pain that is associated with warmth and tenderness, and in the case of unilateral disease, the enlargement or swelling of one limb versus the other.

Well’s scoring has been validated in several studies in the risk stratification for the development of DVT. The use of this scoring system has also been established as a valid pretest tool for risk stratification in trauma patients in one other study (9), however, it has also been shown to be less reliable in predicting inpatient care when compared to outpatient literature (10).

In our study, we report an overall incidence of DVT of 10.3%. We compare this with one study by Hendwood et al. (11), which have studied the incidence of DVT in neurosurgical patients on dual modality DVT prophylaxis which documented an overall rate of 9.7%. This suggests that the rate of DVT in our centre is only marginally higher despite single mechanical modality for DVT prevention.

When reviewing the brain tumours within our study population, the most common diagnosis was brain metastases (*n* = 16), meningiomas (*n* = 8) followed by GBM (*n* = 7). One study by Sawaya et al. (12), gave an incidence of DVT in Meningiomas patients to be 72%, and glioblastomas and metastases incidences of 60% and 20%, respectively. In the brain tumour group, meningiomas, in keeping with other studies, has the highest risk, however, the overall rates of DVT in these categories are lower than these figures. We attribute this to the fact that in our centre, virtually all these cases have come under an elective list, are overall in better pre-operative health and are subjected to a much more aggressive early ambulation and physiotherapy in the gymnasium with the rehabilitation department. We were unable to conclusively make a comparison of the DVT rate between the various brain tumours as well as the cranial versus spine cases, as the sample size was too small.

With regards to D-dimer screening values, our study showed that of the total number of positive ultrasound Doppler findings (*n* = 33), only 1 patient (*n* = 1) had a screening value of less than 4 mg/L. When comparing this to the D-dimer value from the study by Prell et al. (6) (value cut off point of 2 mg/L), we can make suggestions for the value at our centre to be higher- considering a level of, at least 2.5 mg/L.

We had in our study the intention to collect more details from our participants such as the use of oral contraceptives, smoking and level of physical activities or regular exercise, however, where many of the patients who were unable to provide the answers themselves such as in the case of trauma, family members were uncertain of such details.

The number of days in bed were grouped into a mean of 6, 13.5 and 16.5 days and as expected, the longer the patient remained in bed, the higher the risk of developing DVT.

Our study revealed that the Well’s scoring and the number of days in bed had remained the main factors that increased the risk of DVT development. Well’s scoring is not documented routinely for the patients in our neurosurgery unit, accordingly, using the findings of this study the suggestion to make the Well’s score charting as standard for all patients, to be included in the observation charts, has been made. This can help the department identify those who are at greater risks and to guide the neurosurgeon on considering additional modalities of DVT prevention, namely adding a pharmacological agent on a case to case basis, weighing in between the benefits versus the risks.

Of all the patients who developed DVT, only one patient had passed away, however the cause of death was sepsis rather than as a result of the DVT itself.

We would like to declare than there were no sponsors involved and no gratuities were obtained from conducting this study.

## Conclusion

In our study of the factors associated with DVT in neurosurgery patients and overall incidence in surgically operated cases, we found that only the Well’s score and the number of days in bed were significant predictors of the risk for developing DVT when other confounders were adjusted. Those who were in the high risk groups of the Well’s score were at a significantly higher risk of developing DVT, and with every passing day a patient remained non-ambulatory, the risk of DVT increases as well. Despite the use of mainly mechanical and single modality prophylaxis, the rate of occurrence was in fact fairly similar to other centres which used dual modality prophylaxis; suggesting that single modality can be considered for most cases, and advocating dual modality is for high risk cases only.

## Figures and Tables

**Table 1:** Characteristics of study population (*N* = 320)

Characteristics	*n*	(%)
Age, years*		
Mean (SD)	46.00 (14.82)	
Minimum, maximum	18, 65	
**Ethnicity:**		
Malay	204	(63.8)
Chinese	82	(25.6)
Indian	25	(7.8)
Others	9	(2.8)
**Gender:**		
Male	212	(66.3)
Female	108	(33.8)
Body mass index (BMI)*		
Median (IQR)	24.32 (22.72–27.36)	
Minimum, maximum	18.22, 48.89	
**Type of surgery:**		
Cranial	304	(95.0)
Spinal	16	(5.0)
**Nature of surgery:**		
Elective	78	(24.4)
Emergency	242	(75.6)
**Well’s score:**		
High risk	40	(12.5)
Low risk	280	(87.5)
**Type of central venous access:**		
None	48	(15.0)
Femoral	233	(72.8)
Internal jugular	1	(0.3)
Subclavian	38	(11.9)
**DVT prophylaxis:**		
None	6	(1.9)
Compression stocking	125	(39.1)
Heparin	23	(7.2)
Pneumatic cuff	166	(51.9)
**D-dimer screening result:**		
Positive	133	(41.6)
Negative	187	(58.4)
**Doppler ultrasonography result:**		
Positive	33	(10.3)
Negative	100	(31.3)
Not applicable	187	(58.4)

SD = standard deviation, IQR = interquartile range, reported as 25th percentile–75th percentile,

* continuous data

**Table 2:** The association between characteristics and DVT diagnosis using Doppler ultrasonography

	Positive (*n* = 33) *n* (%)	Negative (*n* = 100) *n* (%)	Not available (*n* = 187) *n* (%)	Test-statistics (df)^a^	*P*-value^a^
**Age group:**					
≤45 years	14 (42.4)	43 (43.0)	84 (44.9)	0.14 (2)	0.934
> 45 years	19 (57.6)	57 (57.0)	103 (55.1)		
**Ethnicity:**					
Malay	21 (63.6)	69 (69.0)	114 (61.0)	-	0.828^b^
Chinese	10 (30.3)	22 (22.0)	50 (26.7)		
Indian	2 (6.1)	6 (6.0)	17 (9.1)		
Others	0 (0.0)	3 (3.0)	6 (3.2)		
**Gender:**					
Male	24 (72.7)	63 (63.0)	125 (66.8)	1.12 (2)	0.571
Female	9 (27.3)	37 (37.0)	62 (33.2)		
**Type of brain tumour:**					
Cranial	32 (97.0)	99 (99.0)	173 (92.5)	6.07 (2)	0.048
Spinal	1 (3.0)	1 (1.0)	14 (7.5)		
**Type of hospital admission:**					
Emergency	24 (72.7)	79 (79.0)	139 (74.3)	0.94 (2)	0.626
Elective	9 (27.3)	21 (21.0)	48 (25.7)		
**Well’s score:**					
High risk	8 (24.2)	6 (6.0)	26 (13.9)	8.36 (2)	0.015
Low risk	25 (75.8)	94 (94.0)	161 (86.1)		
**Type of central venous access:**					
Femoral	24 (72.7)	65 (65.0)	144 (77.0)	-	< 0.001^b^
Internal jugular	1 (3.0)	0 (0.0)	0 (0.0)		
Subclavian	7 (21.2)	6 (6.0)	25 (13.4)		
None	1 (3.0)	29 (29.0)	18 (9.6)		
**Type of DVT prophylaxis:**					
Compression stocking	12 (36.4)	29 (29.0)	84 (44.9)	-	0.006^b^
Heparin	0 (0.0)	12 (12.0)	11 (5.9)		
Pneumatic cuff	19 (57.6)	59 (59.0)	88 (47.1)		
None	2 (6.1)	0 (0.0)	4 (2.1)		
**D-dimer screening:**					
Positive	33 (100.0)	100 (100.0)	0 (0.0)	-	NA
Negative	0 (0.0)	0 (0.0)	187 (100.0)		
No. of days in bed*:					
Median (IQR)	16.50 (8.00–52.75)	13.50 (2.00–34.00)	6.00 (2.00–29.00)	-	0.002^c^
Range	3, 129	1, 92	1, 119		

a Chi-square test,

b Fisher’s exact test,

c Kruskal-Wallis test

NA = Not applicable to analyse since negative D-Dimer screening was used to rule-out the DVT patient IQR = interquartile range, reported as 25th percentile–5th percentile

Range is reported as minimum, maximum value

* Excluding bed bound and passed-away patients

**Table 3:** The association between type of brain tumour, type of hospital admission, Well’s score and number of days in bed with DVT diagnosis outcome by Doppler ultrasonography, using multiple logistic regression

	Positive (*n* = 33) *n* (%)	Negative (*n* = 100) *n* (%)	Adj OR (95% CI) a	*X2*-statistics (df)^b^	*P*-value^b^
**Type of brain tumour:**					
Cranial	32 (97.0)	99 (99.0)	NA	-	-
Spinal	1 (3.0)	1 (1.0)			
**Type of hospital admission:**					
Emergency	24 (72.7)	79 (79.0)	0.97 (0.25, 3.69)	0.00 (1)	0.959
Elective	9 (27.3)	21 (21.0)	1.00		
**Well’s score:**					
High risk	8 (24.2)	6 (6.0)	7.18 (1.12, 45.95)	4.33 (1)	0.037
Low risk	25 (75.8)	94 (94.0)	1.00		
**Number of days in bed**	-	-	1.02 (1.00, 1.05)	4.76 (1)	0.029

a Adjusted for age group, gender, ethnicity, type of central venous access and type of DVT prophylaxis. Type of brain tumour was excluded from the regression since there is only one observation of spinal tumour for each diagnosis group. Total data included in the analysis is 100 (33 cases [25%] of patient with bed bound or passed away were excluded)

b Wald test

Adj OR = adjusted odds ratio; CI = confidence interval; df = degree of freedom; NA = not applicable
